# Tackling Tumour Cell Heterogeneity at the Super-Resolution Level in Human Colorectal Cancer Tissue

**DOI:** 10.3390/cancers13153692

**Published:** 2021-07-22

**Authors:** Fabian Lang, María F. Contreras-Gerenas, Márton Gelléri, Jan Neumann, Ole Kröger, Filip Sadlo, Krzysztof Berniak, Alexander Marx, Christoph Cremer, Hans-Achim Wagenknecht, Heike Allgayer

**Affiliations:** 1 Institute of Organic Chemistry, Karlsruhe Institute of Technology (KIT), Fritz-Haber-Weg 6, Campus Süd, 76131 Karlsruhe, Germany; fabian.lang2@kit.edu (F.L.); hans-achim.wagenknecht@kit.edu (H.-A.W.); 2 Department of Experimental Surgery—Cancer Metastasis, Mannheim Medical Faculty, Ruprecht-Karls University of Heidelberg, Ludolf-Krehl-Straße 13-17, 68167 Mannheim, Germany; mfcontrerasg@gmail.com; 3 Institute of Molecular Biology (IMB), Ackermannweg 4, 55128 Mainz, Germany; m.gelleri@imb-mainz.de (M.G.); jan.neumann@mail.de (J.N.); c.cremer@imb-mainz.de (C.C.); 4 Interdisciplinary Centre for Scientific Computing (IWR), University Heidelberg, Mathematikon B, Im Neuenheimer Feld 205, 69120 Heidelberg, Germany; o.kroeger@opensourc.es (O.K.); sadlo@uni-heidelberg.de (F.S.); 5 Department of Cell Biophysics, Faculty of Biochemistry, Biophysics and Biotechnology, Jagiellonian University, Gronostajowa 7 Street, 30-387 Krakow, Poland; kberniak@gmail.com; 6 Institute of Pathology, Mannheim Medical Faculty, Ruprecht-Karls University of Heidelberg, Theodor-Kutzer-Ufer 1, 68167 Mannheim, Germany; Alexander.Marx@umm.de; 7 Institute of Pharmacy & Molecular Biotechnology, Ruprecht-Karls University of Heidelberg, Im Neuenheimer Feld 364, 69120 Heidelberg, Germany

**Keywords:** chromatin density, nanoscale, tumour cell heterogeneity, microRNAs, metastasis, super-resolution microscopy

## Abstract

**Simple Summary:**

Tumour cell heterogeneity is the most fundamental problem in cancer diagnosis and therapy. Micro-diagnostic technologies able to differentiate the heterogeneous molecular, especially metastatic, potential of single cells or cell clones already within early primary tumours of carcinoma patients would be of utmost importance. Single molecule localisation microscopy (SMLM) has recently allowed the imaging of subcellular features at the nanoscale. However, the technology has mostly been limited to cultured cell lines only. We introduce a first-in-field approach for quantitative SMLM-analysis of chromatin nanostructure in individual cells in resected, routine-pathology colorectal carcinoma patient tissue sections, illustrating, as a first example, changes in nuclear chromatin nanostructure and microRNA intracellular distribution within carcinoma cells as opposed to normal cells, chromatin accessibility and microRNAs having been shown to be critical in gene regulation and metastasis. We believe this technology to have an enormous potential for future differential diagnosis between individual cells in the tissue context.

**Abstract:**

Tumour cell heterogeneity, and its early individual diagnosis, is one of the most fundamental problems in cancer diagnosis and therapy. Single molecule localisation microscopy (SMLM) resolves subcellular features but has been limited to cultured cell lines only. Since nuclear chromatin architecture and microRNAs are critical in metastasis, we introduce a first-in-field approach for quantitative SMLM-analysis of chromatin nanostructure in individual cells in resected, routine-pathology colorectal carcinoma (CRC) patient tissue sections. Chromatin density profiles proved to differ for cells in normal and carcinoma colorectal tissues. In tumour sections, nuclear size and chromatin compaction percentages were significantly different in carcinoma versus normal epithelial and other cells of colorectal tissue. SMLM analysis in nuclei from normal colorectal tissue revealed abrupt changes in chromatin density profiles at the nanoscale, features not detected by conventional widefield microscopy. SMLM for microRNAs relevant for metastasis was achieved in colorectal cancer tissue at the nuclear level. Super-resolution microscopy with quantitative image evaluation algorithms provide powerful tools to analyse chromatin nanostructure and microRNAs of individual cells from normal and tumour tissue at the nanoscale. Our new perspectives improve the differential diagnosis of normal and (metastatically relevant) tumour cells at the single-cell level within the heterogeneity of primary tumours of patients.

## 1. Introduction

One of the most challenging problems in oncology, diagnosis and (personalised) tumour therapy still is the huge heterogeneity of carcinoma cells within tumours of patients with malignant diseases. It is estimated that each patient primary tumour comprises millions of different tumour cells/tumour cell clones that differ in their molecular and functional characteristics. Within this huge heterogeneity of cells in tumours, some cancer cells show rather low, some others high potential to promote cancer progression, to give rise to metastasis, which is still the cause of about 90% of cancer-related deaths [1], or to respond, or become resistant, to classical or novel personalised therapeutic strategies [2,3,4]. This has been shown recently, for example, in our own whole genome sequencing work in which it became apparent that primary tumours and their corresponding metastases originate from a common ancestor clone each, but that metastases still develop further changes already at the genome level as compared to their corresponding primaries, an issue which might impact tremendously therapy response or resistance [5]. Thus, recent years of research, especially in the fields of cancer stem cells, metastasis-initiating cells, single cell analysis [5,6,7,8], and others have illustrated how important it will be for individualised diagnosis and therapy to be able to differentiate cells with different molecular, metastatic, and therapeutic potential already at the stage of the primary tumour. Especially, to improve patient prognosis fundamentally and prospectively, it will be highly important to establish micro-diagnostic tools able to detect, ideally, single tumour cell clones that harbour a high risk to lead to later disease recurrence and metastasis, or particular patterns of therapy resistance within the primary tumour, before these cells give rise to macroscopic relapse. This would enable a new generation of targeted therapy design which is capable of preventing crucial tumour cell clones from spreading, growing, and metastasizing.

Nuclear architecture is crucial for determining the levels of gene activation. However, the extent and means by which chromatin structure alters gene expression are not fully understood yet. Early detection of replication “factories” allowed for a simplified nuclear model comprising heterochromatin (dense, inactive chromatin) and euchromatin (loose, transcription factor-enriched chromatin) [9]. Recently, a more detailed model has been proposed which predicts that the nuclear genome is partitioned into two co-aligned regions, an active nuclear compartment with low DNA density and an inactive nuclear compartment with high DNA density. There is also an interchromatin compartment in this model, which consists of channel-like regions mostly with a very low DNA density [10]. Areas of low density have been proposed to harbour loci of active gene transcription [10,11]. Moreover, studies have already suggested that reprogramming the chromatin status and increasing chromatin accessibility by different molecular means in cancer cells is associated with promoting cancer metastasis [12,13]. Moreover, the use of drugs that help recover normal nuclear architecture features has been linked with the recovery of normal cell phenotype [14].

Thus, diagnostic tools that involve high-resolution options within the chromatin nanostructure might be highly interesting to identify cells critical for progression, metastasis, or therapy response, if these tools could be applied directly to histopathological whole sections already of patient primary tumours and further tissues. This would be critical also since structural differences have been observed between cells (or cell lines) in monolayer cultures and the same cells in tissues [15]. However, up to now, attempts in this context to achieve subcellular imaging of the DNA distribution in tumour cells at the nanoscale with SMLM have been rare. In our own preliminary studies, we started to study chromatin distribution by confocal and SMLM [16], whereby the percentage of compact chromatin calculated from confocal microscopy images proved to differ for cells with different functionality. SMLM data in nuclei from normal colorectal tissue revealed abrupt changes in chromatin density profiles at the nanoscale, features not detected by conventional widefield microscopy. These observations highlight the importance of advancing super-resolution techniques like SMLM as individual diagnosis tools of single cell heterogeneity [17], as well as the enormous potential that this can have for answering biological/medical questions such as diagnosing individual cells within an individual patient with different metastatic or therapy response potential.

In the diagnosis of metastatically capable cells, microRNAs (miRs) could play a highly interesting role as well since many of these have been shown, by us and others, to be critical players in the metastatic process [18,19,20,21,22]. MiRs have an average length of 22 nucleotides and belong to the class of small non-coding RNAs. They bind with their seed sequence to the 3′ untranslated region (UTR) of their target and then degrade, or block translation of, the target mRNA [23]. More recently, additional nuclear functions of miRs have been elucidated, which include post-transcriptional and transcriptional gene silencing, and the transcriptional activation of genes [24], this potentially links miR-intracellular diagnostics to chromatin diagnostics. Specific aberrant miRNA expression profiles have been identified in different cancer types including colorectal cancer, especially as pro-or antagonists of metastasis at several instances. Towards this end, miR are not only direct regulators of mRNAs for oncogenes, tumour suppressor genes, or metastatically relevant genes, but also are able to, for example, prime metastatic niches systemically via being spread within exosomes by primary cancer cells [25,26,27,28]. Therefore, miRNA imaging could play an important role in cancer diagnostics but is a challenge for imaging methods due to the short length and homologues. Several miRNA imaging methods have been developed mainly based on in situ hybridisation (ISH) [29], nucleic acid amplification [30], northern blotting [31], or microarray [32]. These methods have been improved with regard to sensitivity, specificity, and imaging over the past years [33]. Nevertheless, detection of miRNAs with microscopic methods was restricted due to the conventional limit of resolution in optical microscopy. With the help of SMLM, we recently overcame this limitation and showed the detection of single miRNA molecules at the superresolution level in single cells of cultured colorectal cancer cell lines [34]. However, successfully establishing this technology for single cell, single molecule subcellular diagnosis in specific resected tissues of patients still remains undone.

Herein, we present the first successful combination of Fluorescence in situ Hybridisation (FISH) with SMLM based chromatin nanotexture and microRNA analysis in routine-pathology, paraffin-embedded colorectal cancer tissue sections, to characterise cellular heterogeneity in the primary tumour tissue of colorectal cancer patients. We believe that this technology could be interesting for the future to identify tumour cell clones of, for example, higher individual metastatic potential or therapy resistance, given their chromatin texture and intracellular distribution and number of metastatically relevant microRNA molecules.

## 2. Material and Methods

### 2.1. Ethics Approval and Colorectal Tissue Samples

Formalin-fixed, paraffin embedded (FFPE) tissue blocks were generously provided by Prof. Alexander Marx (Pathologisches Institut, UMM/Mannheim Medical Faculty of Heidelberg University, Germany). Anonymous colorectal carcinoma and paired corresponding normal (colorectal) tissue blocks were available from the archival material of the same patients. Approval by the institutional ethics board (Ethics board II at UMM, approval no. 2017-806R-MA) was granted to AM, waiving the need for informed consent for this retrospective and fully anonymised analysis of archival pathological samples.

FFPE blocks were cut using a Leica RM2255 fully automated rotary microtome. The first few sections were discharged and 10 µm thick sections were collected on top of previously cleaned coverslips (No. 1.5H, Paul Marienfeld, Lauda-Könishofen, Germany) from a 37 °C water bath. Coverslips were transferred to a 65 °C oven and left there for a minimum of 30 min. Dried slices were kept at 4 °C for not more than a month before further processing (FISH, confocal microscopy or SMLM).

### 2.2. Fluorescence In Situ Hybridisation (FISH) on Colorectal Tissue Sections

Tissue sections on coverslips were accommodated in a custom-made Teflon coverslip holder and placed for 5-min steps in the following solutions: Roticlear, 1:1 Roticlear: 100% EtOH, ethanol series (100%, 95%, 70%, 50%, 25%), briefly in DI water and PBS. For the antigen retrieval step, 2 mL of a 1 µg/mL of proteinase K (NEB, Ipswich, MA, USA) in 50 mM Tris buffer was added per tissue section in a 6-well plate (15 min, RT, gentle stirring). Two washes followed, one with 0.1% Tween-20/PBS and the second one with PBS. Samples were permeabilised in a 0.5% Triton-X 100/PBS solution (1 h, RT, gentle stirring), followed by two PBS washes and immediate dehydration in ethanol solution steps of increasing concentration (25%, 50%, 70%, 95%, 100%), followed by air drying (5 min each). A boundary was put around the tissue samples using Picondent Twinsil (Picodent, Wipperfürth, Germany). Subsequent volumes were added to an extent that the entire sample was covered (maximum 100 µL). Hybridisation solution (50% *v/v* formamide, 0.75 M NaCl, 75 mM Na-Citrate, 50 mg/mL heparin, 0.5% *v/v* Tween-20) was added to each sample (15 min, 37 °C). Prior to hybridisation, miR probes were denatured at 80 °C for 5 min. The probes were diluted in hybridisation buffer to 200 nM and added to the samples. Hybridisation was performed overnight at 37 °C. Stringent washes were performed twice in 0.2× SSC (10 min each, RT, gentle stirring), for 5 min each at RT under gentle stirring.

### 2.3. DNA Probe Synthesis and Characterisation

DNA sequences complementary to the four metastatically relevant miRNAs miR-135b, miR-210, miR-21 and miR-31 studied in this work, as well as a positive control (sequence against U6 snRNA) and negative control (scrambled sequence) were synthesised. The most central thymidine was exchanged for a 2′-*O*-propargyl-uridine (cU) (building block is commercially available from GlenResearch) to enable the post-synthetic modification with fluorophores via copper-catalysed azide-alkyne cycloaddition (CuAAC). The solid-phase phosphoramidite synthesis was performed with an H6 DNA/RNA synthesizer (K&A Laborgeräte, Schaafheim, Germany), using standard protocols. For the incorporation of the cU-phosphoramidite, the reaction time of the coupling step was increased to 4 min. After successful synthesis and cleavage from the solid phase with 24% ammonia solution overnight at 60 °C, the synthesised DNA probes were modified in two separate sets with Cy5-azide and AF488-azide [35]. Cy5 is a well-established fluorophore in confocal microscopy and was chosen for the proof of concept. AF488 has a better blinking behaviour than Cy5 which makes it suitable for superresolution microscopy. After modification, the oligonucleotides were purified via reversed-phase HPLC with a Thermo Fisher UltiMate 3000 (Thermo Fisher Scientific Inc., Waltham, MA, USA) (0–40% acetonitrile in 50 mM ammonium acetate buffer). Spectroscopic measurements were performed with solutions of 2.5 µM FISH-probe, 2.5 µM corresponding RNA, 50 mM Na-P_i_ buffer, and 250 mM NaCl in water at pH 7. Absorption spectra were measured with a *Varian Cary 100* UV/Vis-spectrometer, and fluorescence spectra were measured with a *HORIBA FluoroMax 4* spectrofluorometer. The used excitation wavelengths were λ_exc_ = 647 nm for Cy5 probes and λ_exc_ = 488 nm for AF488 probes.

### 2.4. Confocal Microscopy on Colorectal Tissue Sections

Imaging of the tissue sections was carried out on a Leica TCS SP5 STED microscope (used in confocal mode) using either a 40×/1.1 or a 63×/1.2 water immersion objective. Sytox Orange was excited by using a 561 laser. A HyD detector (nm range) was employed to capture the signals.

### 2.5. Chromatin Compaction Quantitative Analysis—Confocal Data

A total of 50 nuclei images of each of the colorectal cell types (normal tissue: epithelial cells of the mucosa; stromal cells of the submucosa, and smooth muscle cells of the L. muscularis layer; carcinoma: epithelial-derived tumour cells) were selected from representative confocal images. For the analysis, the area of each nucleus was estimated using an ImageJ’s macro. Local intensity maxima were acquired in this area. The number of detected maxima in the nucleus was defined as the number of chromatin domains [36]. To determine the local neighbourhood area for each peak, changes in signal intensity distribution on the image of the stained nucleus were taken into account. All image processing was performed by using an ImageJ algorithm [37]. To obtain the dark area of each nucleus (low chromatin density area), the total area of all recognised domains was subtracted from the size of the whole nucleus.

### 2.6. miRNA Signal Quantification and Statistical Analysis

Confocal images of samples on which FISH was performed were analysed using Columbus Software (PerkinElmer, Waltham, MA, USA), to identify the miRNA positive (miR+) cells within the tissue. 20× magnification fields of view from the confocal images were analysed. A visual description of the pipeline can be found in Figure S5. Briefly, nuclei were identified from an input image based on the nuclear dye signal intensities. A contour was drawn estimating the cytoplasm region for each identified nucleus. Finally, a list of cells was obtained and filtered exclusively for cells that had a strong probe signal in the cytosolic area (defined as miR+). A strong probe signal was defined as an intensity in the miR channel above or higher than 5. This cut-off value was chosen given that all the measured intensities in the miR channel on negative control samples that were not incubated with miR-probe, but scrambled sequence instead, were below this value as background signals. Quantified values from this analysis are presented as the percentage of miR+ cells in a given tissue area (field of view).

Statistical analysis was performed with R statistical software (version 4.0.0, The R Foundation for Statistical Computing, Vienna, Austria). An unpaired one-sided Wilcoxon test (*p* ≤ 0.05 was considered significant) was used to compare the abovementioned quantified values between cancer tissue and its matched normal counterpart.

### 2.7. SMLM on Colorectal Tissue Specimen

2D single molecule localisation microscopy (SMLM) was performed on an SR GSD setup (Leica Microsystems CMS GmbH, Wetzlar, Germany), based on an inverse widefield microscope DMI AF6000 equipped with an oil immersion objective (160×/1.43). Fluorescent emission was captured and imaged on an EMCCD camera with a pixel size of 100 nm (iXon3 Ultra 897, Andor Technology Ltd., Belfast, UK). Prior to imaging, the tissue sections on coverslips (see colorectal tissue samples) were deparaffinised and rehydrated (see Fluorescence in situ hybridisation on colorectal tissue sections). Samples were permeabilised in 0.5% Triton/PBS for 1 h, covered with 50 nM Sytox Orange in PBS for 1 h, and embedded on freshly prepared GOX/CAT blinking buffer (0.5 mg/mL glucose oxidase (GOX), 40 µg/mL catalase (CAT) and 10% *w/v* glucose, all in PBS) [16]. The coverslip was sealed with a two-part epoxy sealant (Picondent Twinsil, Wipperfürth, Germany) to a glass slide. All the above mentioned steps were executed at room temperature, and three PBS washes were carried out in between steps. Samples were taken to the microscope right after being embedded in the blinking buffer. For Sytox Orange imaging, a 561 nm laser (2.1 mW) and 30,000 frames with 30 ms camera exposure were used. Single molecule reconstructions were generated with ThunderSTORM (Fiji plugin).

### 2.8. Quantitative Analysis (SMLM)

#### Chromatin Density Profiles (Binning Analysis)

To compare the chromatin density distributions between colorectal carcinoma and the corresponding normal tissue nuclei, an in-house R script was used to bin single molecule localisation signals in a 50 × 50 nm^2^ grind. The histograms were visualised in the form of violin plots, showing the frequency of bins with a given number of localisation signals. The R script was fed with the localisation tables obtained from ThunderSTORM (Fiji plugin, Prague, Czech Republic). Briefly, a 2D histogram with a given bin size (50 × 50 nm^2^) was generated using the geom_bin2d function. Then, the value of the number of localisations on each bin was transferred into a table.

### 2.9. Radial Chromatin Density (Ring Analysis)

A region of interest (ROI) was manually drawn, enclosing the nuclear area on each of the reconstructed images generated by ThunderSTORM. An in-house MATLAB (MATLAB version 9.3.0 (2017b), The Mathworks, Natick, MA, USA) code was used to subdivide the cell nucleus into six concentric rings with equal area, and to draw concentric areas following the nuclear contour as a guide [38]. The number of localisation signals in each of these rings served as a basis to calculate the average localisation density per ring.

## 3. Results

Distinctive regions within whole 10 µm tissue sections of human colorectal carcinomas and matched corresponding normal tissues were analysed in a first-in-field attempt with SMLM super-resolution microscopy at the nanoscale, following confocal resolution imaging. In the following, we present results and images for chromatin density within nuclei of carcinoma, normal epithelial, and further cell types in CRC tissue sections, followed by single molecule microRNA subcellular analysis for selective miRs, which have been shown to play significant roles in different aspects of metastasis [18,20,34]. Altogether, we hypothesise that this technology could be a highly supportive tool to decipher molecular (tumour) cell heterogeneity at the single cell level within any individual tissue context.

### 3.1. Chromatin Density of Nuclei from Colorectal Carcinoma and Its Corresponding Normal Tissues

#### 3.1.1. Chromatin Density—Confocal Resolution

Nuclei from individual tissue regions depicted in Figure S1 were analysed independently to demonstrate that nuclei from cells with distinctive functions (benign and malignant epithelial cells, stromal cells including smooth muscle cells from different tissue layers) would lead to distinctive chromatin density profiles. Figure S1 shows the three distinctive regions taken for the analysis: mucosa, submucosa, and the muscularis layer. Nuclei analysed within the colorectal carcinoma specimen corresponded to the tumour areas, regions enclosed in blue (Figure S1A).

**Figure 1:**
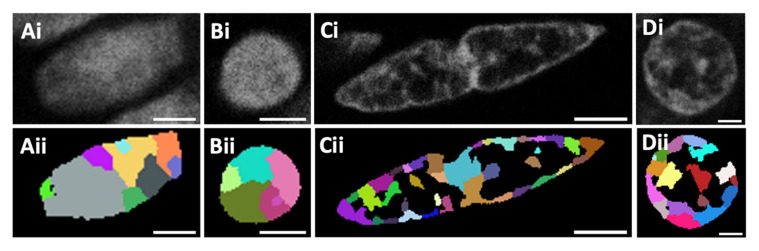
Representative confocal images of human colorectal tissue nuclei stained with Sytox Orange dye. (**Ai**–**Ci**) Images from a normal colorectal tissue sample comprising a nucleus from the mucosa (normal epithelial cell) (**Ai**), the submucosa (**Bi**) and the muscularis (**Ci**) layer. An image from a colorectal carcinoma tissue sample shows a carcinoma cell nucleus from the tumour region (**Di**). Resulting images for each cell type after the segmentation analysis was performed (**Aii**–**Dii**). Scale bars: 3 µm.

**Figure 2:**
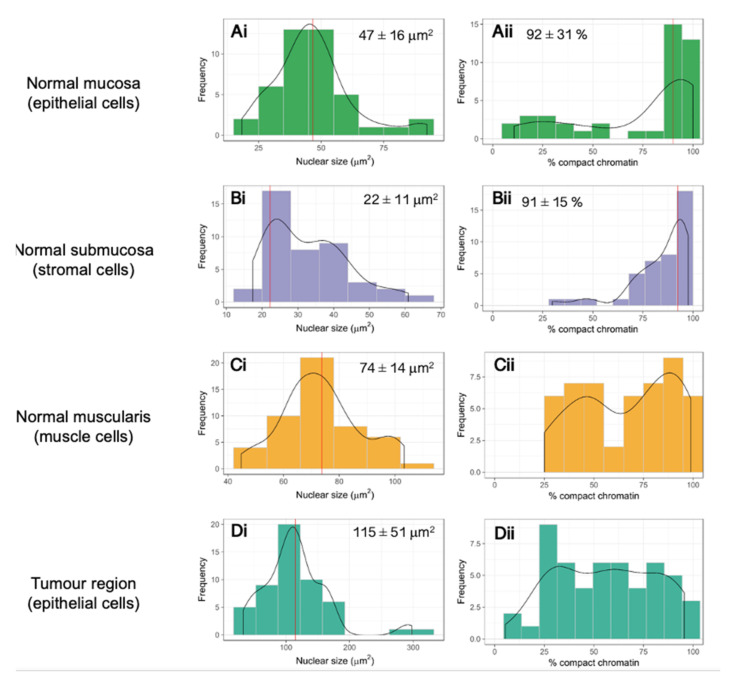
(**Ai**–**Ci**) Nuclear size distribution of normal colorectal tissue cell types: epithelial cells of the normal mucosa (**Ai**), stromal cells (Tunica submucosa) (**Bi**) and muscle cells (Tunica muscularis) (**Ci**). Nuclear size distribution of epithelial nuclei from a tumour area within a representative example of a colorectal carcinoma tissue specimen (**Di**). (**Aii**–**Dii**) Percentage of compact chromatin in the same normal and carcinoma colorectal cell types. Solid red vertical lines correspond to the mode from the data. Reported values are the mode ± SD. For normal distributions, the mode coincides with the mean of the distribution. Distributions in (**Cii**,**Dii**) might correspond to a multimodal distribution (*n* = 50).

To obtain a first overview of the different chromatin density distributions and features between cell types, the human colorectal tissue nuclei were stained with Sytox Orange and imaged with a confocal microscope (for details, see Section 2 Materials and Methods). Different nuclear architecture features were observed between (epithelial-derived) carcinoma and normal epithelial cells, as well as between carcinoma, normal epithelial, stromal cells within the submucosa, and smooth muscle cells within the muscularis layer. For each of these cell types, images of 50 nuclei were selected from the confocal images for quantitative analysis. Segmentation analysis was performed using an ImageJ algorithm (see Section 2 Materials and Methods). The area of each nucleus was classified into either a region of high intensity (compact chromatin) or a region of low intensity (loose chromatin). Figure 1 shows representative confocal images and the results after performing the segmentation analysis. Figure 2 shows the nuclear size distributions and percentages of chromatin compaction for the different cell types from a human colorectal carcinoma and the corresponding normal tissue specimen.

To characterise and quantify the differences in chromatin density regions between cells with different functionality (epithelial, submucosa, and muscle) and malignancy (non-malignant epithelial versus carcinoma cells), a quantitative analysis was conducted considering a minimum number of 50 cell nuclei from each of the abovementioned cell types. The two main properties that were analysed were the nuclear size and the percentage of bright nuclear area (coloured domains of Figure 1), which quantifies the chromatin compaction.

Figure 2 shows the sizes of the nuclear cross sections in µm^2^ (Figure 2(Ai–Di)) as well as the quantification of the percentage of compact chromatin per nucleus (Figure 2(Aii–Dii)) in a normal and carcinoma colorectal tissue sample with a thickness of 10 µm. Values shown in the nuclear size distribution graphs correspond to mode values. In the case of a normal distribution, the mode value corresponds to the mean.

Figure 2(Aii–Dii) shows the distribution of compact chromatin percentages in the same types of cells. The vast majority of nuclei from mucosa-derived normal epithelial and submucosal cells had a high percentage (above 90%) of their chromatin compacted (Figure 2(Aii,Bii)). This is in agreement with a publication that showed that specialised somatic cells have denser and more compact chromatin in comparison to, for example, stem cells [39]. In contrast, smooth muscle cell nuclei distribution showed a higher variability and was multimodal (two peaks), so no central value out of a population of 50 nuclei could be reported (Figure 2(Cii)).

The same type of analysis was performed for cells from the corresponding colorectal carcinoma tissue. Here, our attention was focused on cells belonging to a tumour area (Figure S1). These tumour areas were delineated by an experienced pathologist depending on the morphological changes presented by tumour cells, a common current practice in routine pathological diagnosis. Figure 2(Di) shows the size of the nuclear cross sections in µm^2^ from cells in a tumour area within a carcinoma tissue specimen. Nuclei from these cells showed a considerably larger average nuclear size of 115 ± 51 µm^2^ in comparison to their normal mucosa epithelial cell counterparts, 47 ± 16 µm^2^. Not only their size but also their variability within the distribution was higher for the nuclei of carcinoma cells.

We also observed considerable differences in the distribution showing the percentages of chromatin compaction (Figure 2(Aii, Dii)). Nuclei from carcinoma cells in the primary tumour CRC tissue (Figure 2(Dii)) showed a broad distribution, covering cells with values as low as 5% and as high as 96%. In contrast, nuclei from normal mucosa epithelial cells (Figure 2(Aii)) showed a narrow distribution around a mode of 92%.

#### 3.1.2. Chromatin Density at the Nanoscale

**Figure 3:**
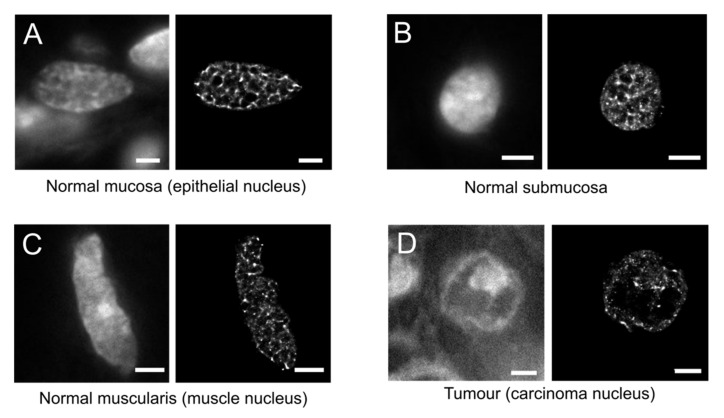
Representative SMLM images of normal colorectal cell nuclei (normal epithelial cell of normal mucosa, submucosa-derived stromal cell, smooth muscle cell within muscular layer) within normal colorectal tissue specimen (**A**–**C**), and of a carcinoma cell nucleus (**D**). Widefield images (**left**) and SMLM reconstructions (**right**) of nuclei imaged from human colorectal tissue sections. Scale bars: 3 µm.

Colorectal tissue samples were stained with Sytox Orange, imaged, and the super-resolution data processed as described for SMLM in Materials and Methods. Figure 3 shows representative images of the super-resolution reconstruction images (right) alongside the widefield images of nuclei from either carcinoma cells of the tumour region within a colorectal carcinoma tissue (D), or from normal epithelial, submucosa-derived stromal cells, and muscle cells of the muscle layer of corresponding normal colorectal tissue, respectively (A–C).

The total number of blinking signals for each one of the reconstructed images shown in Figure 3 is 0.98 × 10^6^ (A), 0.55 × 10^6^ (B), 1.34 × 10^6^ (C), and 0.16 × 10^6^ (D), respectively. Tissue background and signals coming from neighbouring tissue nuclei were present but did not interfere with the measurements, since the background signal within the nuclear region was negligible. Tumour regions within cancer tissue samples still had a higher cell density compared to the corresponding normal colorectal tissue, and therefore, the background signals were higher and the number of blinking events lower. Therefore, to isolate the signals coming exclusively from the nuclei of interest, regions of interest (ROIs) were manually drawn on the widefield images, transferred to the reconstructed images, and only localisation events within these ROIs were considered for downstream analysis.

**Figure 4:**
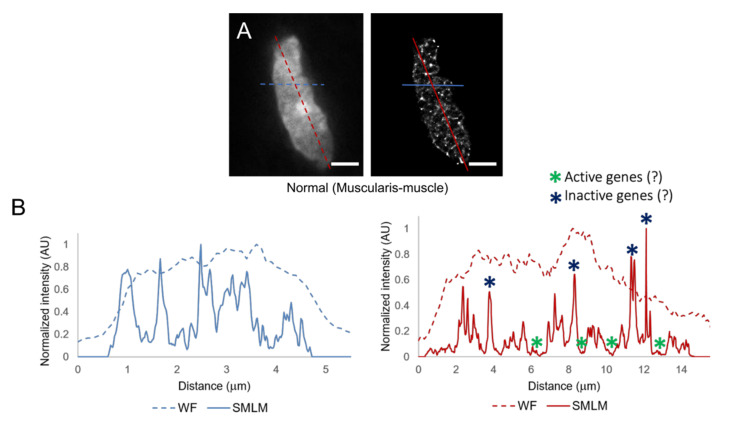
Chromatin density profiles of a normal colorectal muscle nucleus. (**A**) Widefield image and its super-resolved (SMLM) reconstruction. (**B**) Comparison of intensity profiles taken from the widefield image (dotted lines) and the SMLM reconstruction (solid lines). Location of active/inactive genes is hypothesised. Scale bars: 3 µm.

Figure 4 shows the chromatin density profiles of the muscle nucleus showed in Figure 3C. Two profile lines, a vertical (red) and a horizontal (blue), were drawn in both the widefield image (dashed lines) and the super-resolution reconstruction image (solid lines). Figure 4B shows the intensity profiles (distance vs. intensity) for each one of the profile lines. Intensity values correspond to the widefield image for the dashed lines, and to the super-resolution reconstruction for the solid lines.

Strong differences in chromatin density changes can be observed when comparing the widefield profiles with the super-resolved ones. There is a similar trend followed by the two profiles for each one of the graphs in Figure 4B. However, the intensity profiles from the widefield images are not able to detect very low density chromatin regions or regions where the chromatin is highly condensed (very strong intensity signals). The location of active genes (in low density and hence more accessible chromatin regions) and inactive genes (in high density chromatin regions) is hypothesised [36,40,41].

##### The Heterogeneity of Chromatin Density in Human Colorectal (Cancer) Tissue

**Figure 5:**
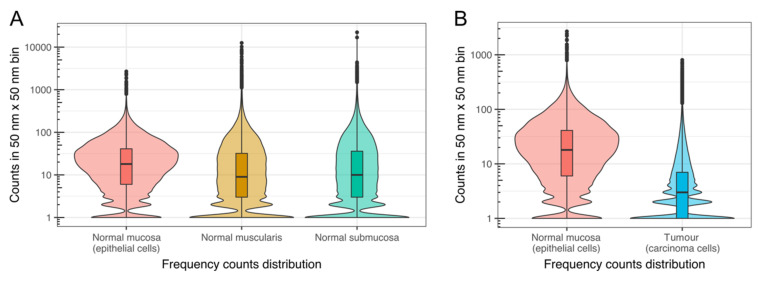
Violin + box plot representations of the localisation signal histograms for nuclei from different regions. A side-by-side comparison is shown between nuclei from (**A**) normal epithelial cells of the mucosa, cells of the submucosa, and of the muscularis layer, and (**B**) between normal epithelial cells of the normal mucosa, and carcinoma cells within the mucosa of a tumour sample. Binning analysis was done in the super-resolved reconstruction images, and signature chromatin density profile distributions were found for each cell type. Horizontal lines in each box plot represent the median value of each one of the distributions. Each distribution is the combination of the replicates (7 for normal submucosa, 2 for normal mucosa, 3 for normal muscularis and 4 for carcinoma cells in the tumour region) for each cell type. Plots of all the replicates from each cell type can be found in Supplementary Figure S2. The horizontal black lines in the middle of the box plots represent the median for each one of the distributions. The values are: mucosa-derived normal epithelial cells 18, muscle cells 9, submucosal cells 13, carcinoma cells 3.

Given that strong differences in the super-resolved reconstruction (SMLM) images were observed when comparing colorectal carcinoma nuclei with the corresponding ones of epithelial cells in normal colorectal tissue, different ways of quantifying and visually presenting the super-resolution data are shown. Figure 5 shows the chromatin density profile distributions for all of the different nuclei analysed. For normal colorectal tissue, data are shown from epithelial, muscle cell and submucosa nuclei within these regions in normal colorectal tissue (Figure 5A). Additionally, a comparison of data from carcinoma cell nuclei (tumour region) versus normal mucosa epithelial cells are shown (Figure 5B). Distributions from Figure 5 were obtained as described in the SMLM—Binning section of Materials and Methods. The distributions are represented as a combination of violin and box plots. The *y*-axis in Figure 5 is a logarithmic scale of the number of blinking events (counts) in a 50 × 50 nm^2^ bin. The *x*-axis quantifies the frequency of a given count number in a bin. It can be observed that, for all types of cells except for the mucosa-derived normal epithelial cells, the big majority of bins had only one event. This was more strongly observed in the carcinoma nuclei than in all of the cell types from the normal colorectal tissue. In contrast, outlier bins (black dots in Figure 5) with very high numbers of counts were detected in all of the different cell types measured. The horizontal black lines in the middle of the box plots of Figure 5 represent the median for each one of the distributions. As can be seen from the figure, the carcinoma nuclei had the lowest median value of all (value of 3), suggesting their low density chromatin areas are more abundant than for all of the normal colorectal cell types in all of the tissue regions investigated.

##### Radial Chromatin Density

To further investigate the chromatin density differences between carcinoma cells and normal cells, we applied the chromatin radial density analysis. The radial density analysis allows visualizing density differences across the nucleus by measuring densities inside rings, starting from the centre of the nucleus to the periphery of the nucleus.

**Figure 6:**
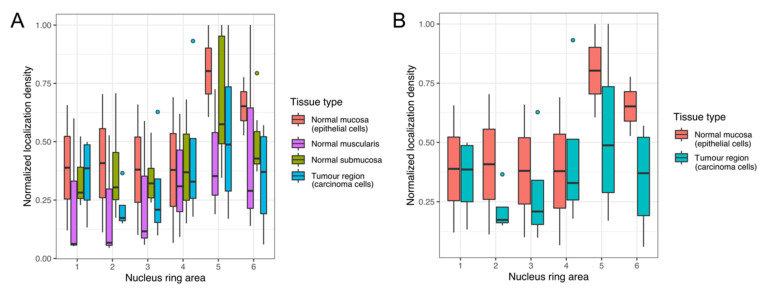
Box plot representations of the radial chromatin density analysis. Box plots show densities measured in rings of equal area from the nuclear centre (Nucleus ring area 1) to the nuclear periphery (Nucleus ring area 6) for cells taken from normal mucosa, normal muscularis, normal submucosa, carcinoma cells from the tumour region (**A**), and, for better comparison, from epithelial cells of normal mucosa and carcinoma cells of the tumour region (**B**). Horizontal lines in each box plot represent the median value of each one of the distributions.

Figure 6 shows the box plot representation of the chromatin radial density analysis for nuclei taken from the epithelial, muscle cell, submucosa, and tumour regions (Figure 6A). Additionally, a comparison of data from carcinoma cells of the tumour region versus normal epithelial cells is shown (Figure 6B).

It can be observed that the chromatin density shows a high variability across individual nuclei and across cell types taken from different regions. For all of the cell types, chromatin densities are highest in the outer rings, indicating a trend to higher chromatin densities close to the nuclear periphery. The centre regions show lower chromatin densities.

Compared to the normal cells, carcinoma cell nuclei taken from the tumour region show a pronounced variability. This becomes evident when looking at the number of outliers. Whereas within the normal cells, only one outlier is present for the submucosa region in the outmost ring, the carcinoma cell distribution shows multiple outliers, suggesting an increased variability within these cells.

### 3.2. miRNA Detection on Human Colon Tissue Sections

In the following, we present our work on the stepwise development of single cell, single molecule microRNA analysis for selective, metastatically relevant miRs to be applicable to tissue sections of human colorectal carcinomas.

#### 3.2.1. DNA Probe Synthesis and Characterisation

**Figure 7:**
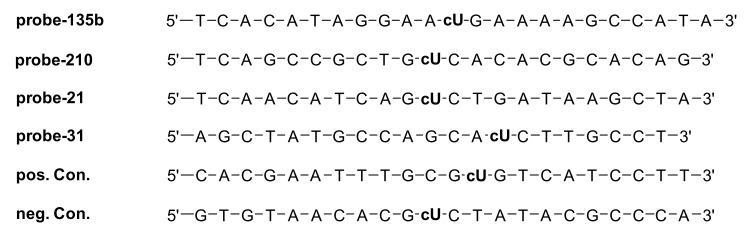
DNA sequences of the synthesised miRNA probes; cU = 2′-*O*-propargyl-uridine was modified with Cy5 (first set of probes) and AF488 (second set of probes).

After synthesis, the commercially available Cy5 dye was incorporated in all probes of interest (as described in Materials and Methods), and the resulting probes were used for the first step of confocal measurements. Probes with AF488 dye were used for single molecule measurements. Figure 7 lists all the sequences of the probes, the miRNA each one targets, and the positive and negative controls used.

Spectroscopic measurements were performed to characterise the optical properties of the synthetic single stranded DNA probes and as annealed hybrids with their target RNA sequences (of the same length, Figure S3). The excitation wavelengths for the fluorescence measurements were 647 nm for Cy5 and 488 nm for AF488, respectively, to fit the laser in the later microscopic experiments. In all cases, the variations in UV/Vis absorption and fluorescence of the DNA probes were rather small, showing that the optical properties do not significantly depend on the individual sequences around the site of fluorophore modification. The Cy5 modified probes showed a slight decrease in fluorescence due to double strand building with the complementary miRNA targets, whereas the AF488 modified probes show a small increase of the fluorescence in the double strand except for the miR-135b probe. The latter probe showed a small decrease that can be explained by the hypochromic shift in absorption.

#### 3.2.2. miRNA Signal Detection of Human Colorectal Tissues

For every set of confocal images, the background signal was subtracted (tissue with no staining) from the images. As positive control, a U6snRNA probe was used, and a scrambled sequence probe served as negative control (Figure S4).

**Figure 8:**
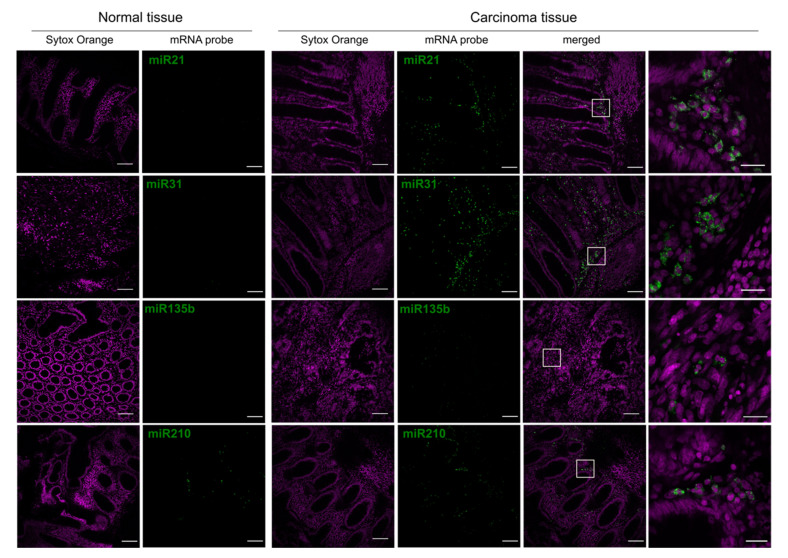
Representative confocal images of FISH performed at normal and carcinoma colorectal human tissue samples with various pro-metastatic miRNA probes: miR-21, miR-31, miR-135b, and miR-210. Green signals correspond to the Cy5 on the miRNA probe, and purple signals represent the nuclear stain by Sytox Orange. The last column shows magnifications of the areas delineated in white rectangles within the second last column (merged images of carcinoma tissue). The brightness range (0–255) of images with miR signals (except the last column) was re-set to 5–100 for this figure alone to enhance the contrast. Quantification of miRNA signals can be found in Figure 9. Scale bars: 100 µm. Scale bars for the last column: 20 µm.

miRNA probes with different fluorophores were synthesised. Amongst these, the set of probes conjugated with Cy5 performed best since their emission spectrum is furthest away from the tissue autofluorescence emission, which was stronger in the 488 channel. Figure 8 shows representative confocal images of normal and carcinoma colorectal human tissues. Images were taken in the mucosa area of each of the tissue samples. Green signals correspond to the miRNA probes conjugated with Cy5, whereas purple signals represent the nuclear DNA stained with Sytox Orange.

Images from Figure 8 confirmed that the four miRNAs imaged here show a higher number of molecules in carcinoma cells of CRC cancer tissue, in contrast to a rather low or absent expression in cells of the normal tissue. This is evident for miR-21 and especially miR-31. For miR-135b, the signal intensity in the carcinoma tissue is lower, but still present in comparison with miR-21 and miR-31. For miR-210, there is some miRNA signal coming from the normal tissue in contrast to what was observed for the other miRs. Still, however, the overall miRNA signal is higher in the carcinoma tissue.

The last column from Figure 8 offers a more detailed view of the miRNA signals. In these zoomed images, miRNA signals appear to be located preferentially in the cytoplasm, close to the nuclear periphery, however, other subcellular locations such as nuclear ones cannot be excluded.

##### miR Positive Cell Fractions

To have a better overview of the FISH results, Columbus Software (PerkinElmer) was used to quantify the number of miR positive (miR+) cells in a given field of view (for a description of the miR+ cells detection pipeline see Section 2 Materials and Methods and Figure S5). miR+ cells were defined here as cells with a high miRNA probe intensity signals located in their cytoplasm. High intensity was defined as an intensity value in the miR channel equal to or higher than 5. Quantification analysis using Columbus Software resulted in a list of miR+ cells with an intensity value representing how strongly a given miRNA signal was observed in each cell’s cytoplasm region. This analysis included miRNA negative (miR-) cells as well, which presented a probe intensity lower than 5 which was comparable to signals detected in the background (see Section 2 Methods).

**Figure 9:**
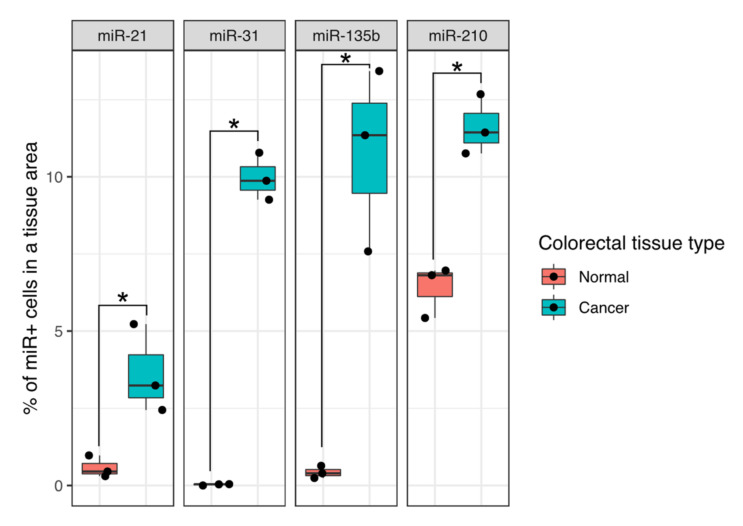
Quantification of the fraction of miR+ cells in normal and colorectal carcinoma human tissues in which FISH was done for four miRNAs that have been shown to be significant players in metastasis. Percentage values shown are with respect to the total cell number in a 20× magnification field of view. Unpaired one-sided Wilcoxon, * *p* ≤ 0.05.

Figure 9 shows the percentage of miR+ cells according to the definition above in 20× magnification fields of view. For each analysed field of view, the total number of cells was quantified (as shown in Figure S5); this value represented 100%. A subset of these cells, the miR+ cells, is shown as a percentage for each of the four miRNAs analysed: miR-21, miR-31, miR-135b, and miR-210.

For all of the four miRNAs analysed, the miR+ cell tissue fractions were always higher in comparison to corresponding normal tissue, which matches the published literature on these miRs including our own publications [18,20,26,34]. Differences between normal and tumour tissues were significant in all four cases.

##### miRNA Signal Detection at the Nanoscale in Human Tissue Samples

**Figure 10:**
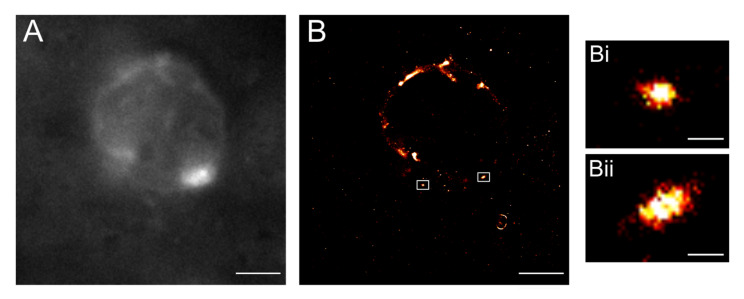
SMLM of miR-21 aggregates in the nuclear area of a carcinoma cell within a human colorectal cancer tissue sample. Smaller blinking events were detected in the cytosolic area as well. (**A**) Widefield and (**B**) super-resolved reconstruction images. Two ROIs are highlighted in white (**Bi**,**Bii**) showing miR21 aggregates. Scale bars 3 µm. Insets scale bars 200 nm.

Based on the previous confocal and FISH analysis, a first-in-field imaging attempt of miRNAs was performed at the single-cell, single-molecule level in routine colorectal cancer patient whole tissue sections. To select miR+ single cells for later SMLM, FISH with a miR-21 probe conjugated with AF488 was conducted on a colorectal carcinoma tissue sample. This fluorophore had to be chosen because of its blinking capabilities in pyranose oxidase embedding buffer, with the disadvantage that its emission coincided with the fluorescence blinking frequency in cases when colorectal tissue presented a strong level of autofluorescence. In spite of this difficulty, SMLM images were taken successfully for carcinoma tissue samples incubated with the miR-21 probe. MiR-signals, and vesicle-like structures containing miR-21 signals, tended to accumulate in the periphery of the nucleus as already shown in confocal microscopy and FISH (see above); in addition, besides some cytoplasmic signals, they also showed localisation signals that seemed to be located inside the nucleus (Figure 10).

Figure 10 shows the representative example of a single-cell nucleus of a human colorectal cancer tissue sample. Figure 10A shows the conventional widefield image of the nucleus, Figure 10B the super-resolved reconstruction image. Aggregates of considerable size of miR-21 signals were detected in the peripheral nuclear area. MiR-21 smaller signals, located in the cytosol, were detected as well (Figure 10B). The two small insets (white rectangles) where miR-21 signals were found to be located in rounded shapes are enlarged (Figure 10(Bi,Bii)). Diameters of the structures in which the miR-signal was condensed were measured along and across (140 × 110 nm for Bi and 240 × 120 nm for Bii, respectively). This visually confirms previous evidence that mature miRNAs, besides a well-described localisation in the cytoplasm and within exosomes [34,42,43,44] are located inside the nucleus and act, for example, like localisation signals for other biomolecules active in chromatin restructuring, nucleolar organisation or in gene silencing/gene activation, chromatin reorganisation in itself having been linked to an increase of metastatic capacity of tumour cells [13,24,45,46]. This, taken together, renders a logical link between analysing chromatin nanostructure and miR-localisation within single cells as first attempts to tackle cancer cell heterogeneity, including the putative metastatic potential of particular cells, within whole tissue sections of individual patient tumours [45].

## 4. Discussion

Our present study demonstrates the feasibility of our single-cell, single-molecule localisation microscopy methodology on whole, 10 µm thick routine patient tissue sections of primary colorectal tumours and corresponding normal tissue for the diagnosis of (changes of) chromatin texture and microRNA subcellular localisation at the nanoscale. We have chosen changes in chromatin nanostructure and particular microRNAs as first diagnostic targets in our attempt to transfer this technology from cell culture/monolayers to an application in human tissue sections, since both of them have been shown to be in the current focus of interest, and of functional importance, for the ability of tumour cells to metastasise [12,13,18,20,34]. Metastasis, as it is well known, is still the most frequent cause of death of cancer patients, and one of the most fundamental unsolved problems for personalised diagnosis and therapy to date is the highly limited ability to recognise specific, metastatically relevant cells or cell clones within the huge context of heterogeneity in primary tumours of patients [47,48,49]. Ideally, diagnostic technologies are needed that are capable of detecting metastatically relevant cells already in an individual primary tumour of a patient within the first stage of initial diagnosis, before relapse and macroscopic metastasis can arise. This also can be crucial for prospective targeted therapy planning, which then could be specifically designed to include particular molecular changes detected in suchlike individual cells.

In the present study, we used confocal and Single Molecule Localisation Microscopy (SMLM) in the fBALM (fluctuation-assisted Binding-Activated Localisation Microscopy) mode to quantitatively analyse the nuclear chromatin texture of cell nuclei in sections of human colorectal cancer tissues. Already the confocal data (Figure 1 and Figure 2) indicated marked differences between the chromatin texture of nuclei in normal and tumour areas of the tissue sections studied. This heterogeneity, however, became much more pronounced in the super-resolved images (Figure 3, Figure 4, Figure 5 and Figure 6). In this case, the local DNA density varied up to almost two orders of magnitude and revealed a large number of very small and highly compacted chromatin clusters down to the sub-resolution range, in apparent contrast to the widefield images.

Very similar results were obtained previously in SMLM-fBALM studies of single nuclei of various cell lines [16,40,50]. To our knowledge, the results reported here for the first time show that this unexpectedly larger heterogeneity in nuclear chromatin nanotexture is also a prominent feature of cells of human colorectal tissue, especially of carcinoma cells in contrast to corresponding normal epithelial cells and other cells of the colorectal tissue context.

This large heterogeneity of nuclear chromatin distribution is typically not observed in conventional resolution microscopy where a much smoother spatial DNA distribution is obtained. The nanoscale heterogeneity of chromatin texture observed by SMLM-fBALM is in striking contrast with a plethora of nuclear genome models based on sequencing approaches like Hi-C [51,52]. These models, however, are derived from biochemical DNA–DNA interaction probabilities; therefore, the maps constructed from these probability data do not necessarily reflect spatial configurations. The SMLM-fBALM results presented here show that, also in nuclei of human colorectal tissue, the spatial distribution of DNA is substantially more heterogeneous than that observed by conventional resolution microscopy: The typically relatively smooth DNA distribution changes imaged with conventional methods were resolved by SMLM into a large number of highly compacted small chromatin domains, interspersed within a large space of low DNA density.

These results are difficult to reconcile with the typical interaction map derived of models cited above, but appear to be fully compatible with the basic tenets of the ANC-INC (Active/Inactive Nuclear Compartment) model, postulating the importance of chromatin density-related accessibility constraints for transcriptional regulation [10,36,41]. In this model, silent gene domains are compacted to such a level of DNA density that the diffusion of macromolecular complexes to target sequences in their interior may be restricted or even excluded. On the other side, transcriptionally competent gene domains are characterised by a low DNA density to enhance accessibility. Previous results obtained with SMLM-fBALM in a mouse cardiomyocyte cell line (HL-1), using YoYo-1 as a DNA stain, also indicated small compacted clusters (around 50–60 nm in diameter) interspersed within a large space of low DNA density. Furthermore, transcriptionally competent small domains were almost exclusively observed within the low density compartment only [40]. The existence of small, highly compacted chromatin domains is also in line with previous observations obtained by other methods of super-resolution microscopy [17,50].

Using Fluorescence in situ hybridisation (FISH) with Oligopaint probes synthesised on the basis of sequence information, it has become possible to directly compare Hi-C data with super-resolved microscopic observations [53]. Other recent observations also favour the concept that nucleosomes are assembled in heterogeneous groups comprising a few kb of DNA, termed nucleosome clutches or clusters (NCs) [39,54]. The acetylation state determines how tightly DNA is compacted within a given NC [55]. The nucleosome cluster concept has been supported by oligo-painting combined with super-resolved fluorescence microscopy [56,57]. While Beliveau et al. [56] used SMLM/STORM with photoswitching dyes, the ORCA method (Optical Reconstruction of Chromatin Architecture) applied by Mateo et al. [57] to Drosophila embryos relied on the sequential imaging of the 3D centre positions of the diffraction limited “spots” produced by each fluorescent probe set in the nucleus; this allowed specific sections within a diffraction-limited volume to be resolved, as in SMLM/STORM, while adding sequence resolution across the domain. A related localisation approach using 3D confocal microscopy and BAC-probes for multicolour painting has previously been reported to explore the 3D nanostructure of a specific small chromatin breakpoint domain in human leukaemia cell nuclei [58]. A comparison of these results with a random walk model indicated highly significant differences between experiment and simulation.

The previous results obtained in a variety of other cell types corroborate the notion that the high nuclear chromatin nanotexture heterogeneity observed here in human colorectal sections is not restricted to individual cells in monolayers but presents a general feature of cells also in tissues. They suggest that the space-time structure of chromatin loops in mammalian cells is generally represented by the existence of small, condensed and dynamic nuclear domains and that these form the essential building blocks for all higher order chromatin structures above the nucleosome level [55]. Altogether, they argue for a DNA density controlled accessibility of macromolecular aggregates to binding sites in the interior of such small compacted chromatin domains. These might exhibit a highly dynamic nanostructure, and we consider it highly likely that such dynamics will also include, for example, significant increases in chromatin accessibility which has been observed during the promotion of metastasis [13]. As a consequence, it may be hypothesised that the accessibility conditions would change accordingly fast, in such a way that the accessibility of a nucleosome in the ensemble centre is dynamically modified with a specific transition frequency, depending on the mechanical elasticity parameters of the ensemble [59]. These dynamics might be different, or enhanced, within the highly heterogeneous nuclei of carcinoma cells which we have found in the present work to be substantially different in size, nanostructure, and distribution of condensed versus relaxed chromatin areas, as compared to corresponding normal epithelia and also other cell types within colorectal tissue. Such a dynamic transition frequency might result in a corresponding modification of the binding probability of transcription factors and, hence, contribute to the fine regulation of gene expression, including genes for miRNAs, or the accessibility for miRNAs and further regulatory molecules to modify gene transcription and chromatin modification [24,45,46]. Since chromatin is an elastic structure, the mechanical properties of such nucleosome clusters might contribute to affect the micro-environmental regulation of genome programs [59,60]. These might include programs to promote migration, invasion and metastasis [5], programs to interact with, and reprogram, the tumour cell microenvironment, to increase the transcription of genes that, for example, contribute to prime metastatic niches, to initiate programs of resistance towards particularly inflicted therapeutic pressure, etc.

In this context, our present work also confirms, within an intact whole tissue setting, that we found blinking signals of particular microRNAs previously discovered to be important players in metastasis, to condense in vesicle-like shapes of the cytoplasm or also in the nucleus. This corroborates previous SMLM super-resolution work we had presented on microRNAs in single cells of cancer cell lines [34] and in which we had demonstrated miR-localisation within exosomes, using an additional exosomal marker. It also corroborates previous findings that miRs can be accumulated in exosomes which, when secreted by tumour cells, can prime metastatic niches in distant organs via systemic spread [25,47,48,49]. Our first-in-field SMLM imaging of microRNAs in the nucleus of a carcinoma cell within the whole tissue context of CRC patients also confirms the functional work of colleagues in recent years who have discovered several nuclear functions of microRNAs in addition to their well-studied cytoplasmic ones as translational inhibitors [24,45,46]. Towards this end, it has been shown that microRNAs can modify, for example, gene expression (activation and silencing of transcription), chromatin remodelling and epigenetics, or nucleolar reorganisation. This can happen via several modes of interaction such as, the binding to nascent RNA transcripts and interactions with promoter and enhancer/silencer regions, or acting within molecular complexes that involve Ago proteins, transcription factors, RNA polymerase II, histone methyltransferase enzymes, and others [24]. Interestingly, one of the interaction models currently postulated suggests that microRNAs might form triple-helical structures with the target DNA, altering chromatin and thus accessibility for transcription factors [24]. In this context, it is interesting to note that, a few years ago, we published a paper showing altered triplex-DNA binding in EMSA studies of colorectal carcinoma as compared to corresponding normal tissue nuclear extracts [61], and it remains to be studied in the future whether carcinoma cell nuclei, in general, show higher triplex formation phenomena as compared to normal cells, which again might be added to an arsenal of diagnostic tools at the nanoscale to tackle single cell heterogeneity.

In summary, we have shown that SMLM super-resolution microscopy is feasible in routine histopathological sections of carcinoma- and matched normal tissues of patients with colorectal cancer, and we anticipate that this will be able to be extended to other tumour types and to further molecules/molecular conditions of interest for single cell heterogeneity diagnosis at the nanoscale. Several future applications for this technological approach can be anticipated, for example, the identification of single tumour cells in the tissue context which show active particular areas of transcription, predictive features at the nanoscale to prepare to become a cancer cell, a particular molecular potential for metastasis, or accessibility to particular therapeutics. We envisage that technologies such as the ones we introduce here could be advanced to automated micro-imaging devices [62], to support and escort pathology and macro-diagnostic imaging fields for a more efficient early differential diagnosis of heterogeneity within individual tumours and patients, up to the successful prevention of cancer progression and metastasis. A highly intriguing additional application of this technology might be to improve the identification and molecular differentiation of single circulating tumour cells (CTCs) in cancer patients [63,64,65], after enrichment, e.g., by flow cytometry and high content conventional resolution micros-copy. On the research side, this would allow to test and image the hypothesis, at the single cell level, that the blood is a pool of nucleic acids derived from different tissue sources and tumour cell subsets with different metastatic capabilities.

## 5. Conclusions

We demonstrate the feasibility of our SMLM methodology on whole, 10 µm thick patient tissue sections of carcinoma patients for the differential diagnosis of heterogeneity in chromatin texture and microRNA subcellular localisation at the nanoscale, and anticipate a promising potential of this methodology to detect, for example, individual carcinoma cells with specific metastatic potential at the molecular level as early as possible in the primary tumour context. For the future, further detailed characterisation of the chromatin density profile and cluster analysis of the images detected with confocal microscopy and SMLM, combined with Oligopainting of cancer-relevant DNA and RNA sequences, is envisaged. These might allow a better understanding of the main differences of chromatin texture, microRNA distribution, and function, in various relevant cell types, in particular, cancer- and metastatically relevant cells. The continuous advancement of this technology, extending to, for example, further molecules to be imaged, to the labelling of particular genes which are active within a given chromatin location, to “multicolour” imaging of several molecular features within the same cells and tissues, and to automated diagnostic systems might open attractive chances to tackle intra-tumour heterogeneity at the earliest possible diagnostic stage. This might contribute to prevent metastatic spread, design therapeutic strategies tailored at the individual heterogeneity of individual tumours, contribute to the development of improved therapeutics with better targeting abilities, or predict the accessibility of particular tumour cell fractions for essential therapeutics.

## Data Availability

The data presented in this study are available in the manuscript and Supplementary Materials. Any further information is available from the authors.

## Supplementary Materials

The following are available online at https://www.mdpi.com/article/10.3390/cancers13153692/s1, Figure S1: (A) Images of human colorectal tissue sections: Section of carcinoma tissue (left) and of the corresponding normal tissue (right). Typical histological layers are depicted side by side for comparison. Blue areas correspond to the tumour areas (drawn by a pathologist) used for further imaging analysis. (B) Enlarged H&E image of a section of carcinoma (left) and normal (right) colorectal tissue. Magnification: 10× carcinoma tissue, 4× normal tissue. A magnification of 4× was used for the latter image with the aim to provide a clear visualisation of the different colorectal layers, Figure S2: Violin + box plot representations of the localisation (SMLM) signal histograms of normal and carcinoma human colorectal tissue nuclei, Figure S3: UV/Vis absorption (A and B) and fluorescence (C and D) of the synthetic DNA probes as single strands, and annealed in hybrids, with their target miRNA sequences; 2.5 µM DNA/RNA, 50 mM Na-Pi buffer, 250 mM NaCl, pH 7, λ_exc_ = 647 nm for Cy5 probes, λ_exc_ = 488 nm for AF488 probes, Figure S4: Positive and negative controls for the FISH staining, Figure S5: Identification of miRNA positive (miR+) cells using Columbus Software (PerkinElmer).

## Abbreviations

ANC-INC: Active/Inactive Nuclear Compartment. CRC: colorectal cancer. fBALM: fluctuation-assisted Binding-Activated Localisation Microscopy. FFPE: Formalin-fixed paraffin embedded. FISH: Fluorescence in situ Hybridisation. HPLC: High Performance Liquid Chromatography. miRs: microRNAs. miR+ cells: miRNA positive cells. NC: nucleosome clutches. RT: room temperature. SMLM: Single Molecule Localisation Microscopy. STORM: Stochastic Optical Reconstruction Microscopy. ROI: region of interest.

## References

[1] Sung H., Ferlay J., Siegel R.L., Laversanne M., Soerjomataram I., Jemal A., Bray F. (2021). Global cancer statistics 2020: GLOBOCAN estimates of incidence and mortality worldwide for 36 cancers in 185 countries. CA Cancer J. Clin..

[2] Mario L. (2005). Colorectal liver metastasis: Towards the integration of conventional and molecularly targeted therapeutic approaches. Front. Biosci..

[3] Gang W., Wang J.-J., Guan R., Yan S., Shi F., Zhang J.-Y., Li Z.-M., Gao J., Fu X.-L. (2018). Strategy to targeting the immune resistance and novel therapy in colorectal cancer. Cancer Med..

[4] Singh M.P., Rai S., Pandey A., Singh N.K., Srivastava S. (2021). Molecular subtypes of colorectal cancer: An emerging therapeutic opportunity for personalized medicine. Genes Dis..

[5] Ishaque N., Abba M.L., Hauser C., Patil N., Paramasivam N., Huebschmann D., Leupold J.H., Balasubramanian G.P., Kleinheinz K., Toprak U.H. (2018). Whole genome sequencing puts forward hypotheses on metastasis evolution and therapy in colorectal cancer. Nat. Commun..

[6] Tieng F.Y.F., Baharudin R., Abu N., Mohd Yunos R.-I., Lee L.-H., Ab Mutalib N.-S. (2020). Single Cell Transcriptome in Colorec-tal Cancer—Current Updates on Its Application in Metastasis, Chemoresistance and the Roles of Circulating Tumor Cells. Front. Pharmacol..

[7] Hervieu C., Christou N., Battu S., Mathonnet M. (2021). The Role of Cancer Stem Cells in Colorectal Cancer: From the Basics to Novel Clinical Trials. Cancers.

[8] Galindo-Pumariño C., Collado M., Herrera M., Peña C. (2021). Tumor Microenvironment in Metastatic Colorectal Cancer: The Arbitrator in Patients’ Outcome. Cancers.

[9] Jackson D., Hassan A., Errington R., Cook P. (1993). Visualization of focal sites of transcription within human nuclei. EMBO J..

[10] Cremer T., Cremer M., Hübner B., Strickfaden H., Smeets D., Popken J., Sterr M., Markaki Y., Rippe K., Cremer C. (2015). The 4D nucleome: Evidence for a dynamic nuclear landscape based on co-aligned active and inactive nuclear compartments. FEBS Lett..

[11] Zhao L., Wang S., Cao Z., Ouyang W., Zhang Q., Xie L., Zheng R., Guo M., Ma M., Hu Z. (2019). Chromatin loops associated with active genes and heterochromatin shape rice genome architecture for transcriptional regulation. Nat. Commun..

[12] Gupta R.A., Shah N., Wang K.C., Kim J., Horlings H.M., Wong D.J., Tsai M.-C., Hung T., Argani P., Rinn J.L. (2010). Long non-coding RNA HOTAIR reprograms chromatin state to promote cancer metastasis. Nature.

[13] Denny S., Yang D., Chuang C.-H., Brady J.J., Lim J.S., Grüner B., Chiou S.-H., Schep A.N., Baral J., Hamard C. (2016). Nfib Promotes Metastasis through a Widespread Increase in Chromatin Accessibility. Cell.

[14] Zink D., Fischer A., Nickerson J.A. (2004). Nuclear structure in cancer cells. Nat. Rev. Cancer.

[15] Lelievre S., Weaver V.M., Nickerson J.A., Larabell C.A., Bhaumik A., Petersen O.W., Bissell M.J. (1998). Tissue phenotype depends on reciprocal interactions between the extracellular matrix and the structural organization of the nucleus. Proc. Natl. Acad. Sci. USA.

[16] Szczurek A., Klewes L., Xing J., Gourram A., Birk U., Knecht H., Dobrucki J., Mai S., Cremer C. (2017). Imaging chromatin nanostructure with binding-activated localization microscopy based on DNA structure fluctuations. Nucleic Acids Res..

[17] Cremer C., Szczurek A., Schock F., Gourram A., Birk U. (2017). Super-resolution microscopy approaches to nuclear nanostructure imaging. Methods.

[18] Asangani I., Rasheed S.A.K., Nikolova D.A., Leupold J.H., Colburn N.H., Post S., Allgayer H. (2007). MicroRNA-21 (miR-21) post-transcriptionally downregulates tumor suppressor Pdcd4 and stimulates invasion, intravasation and metastasis in colorectal cancer. Oncogene.

[19] Mudduluru G., Ceppi P., Kumarswamy R., Scagliotti G.V., Papotti M., Allgayer H. (2011). Regulation of Axl receptor tyrosine kinase expression by miR-34a and miR-199a/b in solid cancer. Oncogene.

[20] Mudduluru G., Abba M., Batliner J., Patil N., Scharp M., Lunavat T.R., Leupold J.H., Oleksiuk O., Juraeva D., Thiele W. (2015). A Systematic Approach to Defining the microRNA Landscape in Metastasis. Cancer Res..

[21] Laudato S., Patil N., Abba M.L., Leupold J.H., Benner A., Gaiser T., Marx A., Allgayer H. (2017). P53-induced miR-30e-5p inhibits colorectal cancer invasion and metastasis by targeting ITGA6 and ITGB1. Int. J. Cancer.

[22] Wen X.-Q., Qian X.-L., Sun H.-K., Zheng L.-L., Zhu W.-Q., Li T.-Y., Hu J.-P. (2020). MicroRNAs: Multifaceted Regulators of Colorectal Cancer Metastasis and Clinical Applications. OncoTargets Ther..

[23] Krol J., Sobczak K., Wilczynska U., Drath M., Jasinska A., Kaczynska D., Krzyzosiak W.J. (2004). Structural Features of MicroRNA (miRNA) Precursors and Their Relevance to miRNA Biogenesis and Small Interfering RNA/Short Hairpin RNA Design. J. Biol. Chem..

[24] Liu H., Lei C., He Q., Pan Z., Xiao D., Tao Y. (2018). Nuclear functions of mammalian MicroRNAs in gene regulation, immunity and cancer. Mol. Cancer.

[25] Garzon R., Calin G.A., Croce C.M. (2009). MicroRNAs in Cancer. Annu. Rev. Med..

[26] Abba M.L., Patil N., Leupold J.H., Moniuszko M., Utikal J., Niklinski J., Allgayer H. (2017). MicroRNAs as novel targets and tools in cancer therapy. Cancer Lett..

[27] Guo Y., Ji X., Liu J., Fan D., Zhou Q., Chen C., Wang W., Wang G., Wang H., Yuan W. (2019). Effects of exosomes on pre-metastatic niche formation in tumors. Mol. Cancer.

[28] Kogure A., Kosaka N., Ochiya T. (2019). Cross-talk between cancer cells and their neighbors via miRNA in extracellular vesicles: An emerging player in cancer metastasis. J. Biomed. Sci..

[29] Obernosterer G., Martinez J., Alenius M. (2007). Locked nucleic acid-based in situ detection of microRNAs in mouse tissue sections. Nat. Protoc..

[30] Chen C., Ridzon D.A., Broomer A.J., Zhou Z., Lee D.H., Nguyen J.T., Barbisin M., Xu N.L., Mahuvakar V.R., Andersen M.R. (2005). Real-time quantification of microRNAs by stem-loop RT-PCR. Nucleic Acids Res..

[31] Lagos-Quintana M., Rauhut R., Lendeckel W., Tuschl T. (2001). Identification of Novel Genes Coding for Small Expressed RNAs. Science.

[32] Thomson J.M., Parker J., Perou C., Hammond S.M. (2004). A custom microarray platform for analysis of microRNA gene expression. Nat. Methods.

[33] Cheng Y., Dong L., Zhang J., Zhao Y., Li Z. (2018). Recent advances in microRNA detection. Analyst.

[34] Oleksiuk O., Abba M., Tezcan K.C., Schaufler W., Bestvater F., Patil N., Birk U., Hafner M., Altevogt P., Cremer C. (2015). Single-Molecule Localization Microscopy allows for the analysis of cancer metastasis-specific miRNA distribution on the nanoscale. Oncotarget.

[35] Schwechheimer C., Doll L., Wagenknecht H. (2018). Synthesis of Dye-Modified Oligonucleotides via Copper(I)-Catalyzed Alkyne Azide Cycloaddition Using On- and Off-Bead Approaches. Curr. Protoc. Nucleic Acid Chem..

[36] Cremer T., Cremer M., Hübner B., Silahtaroglu A., Hendzel M., Lanctôt C., Strickfaden H., Cremer C. (2020). The Interchromatin Compartment Participates in the Structural and Functional Organization of the Cell Nucleus. BioEssays.

[37] Rybak P., Hoang A., Bujnowicz L., Bernas T., Berniak K., Zarębski M., Darzynkiewicz Z., Dobrucki J. (2016). Low level phosphorylation of histone H2AX on serine 139 (γH2AX) is not associated with DNA double-strand breaks. Oncotarget.

[38] Kaufmann R., Lemmer P., Gunkel M., Weiland Y., Müller P., Hausmann M., Baddeley D., Amberger R., Cremer C., Enderlein J., Gryczynski Z.K., Erdmann R. (2009). SPDM: Single Molecule Superresolution of Cellular Nanostructures.

[39] Ricci M.A., Manzo C., Garcia-Parajo M.F., Lakadamyali M., Cosma M.P. (2015). Chromatin Fibers Are Formed by Heterogeneous Groups of Nucleosomes In Vivo. Cell.

[40] Kirmes I., Szczurek A., Prakash K., Charapitsa I., Heiser C., Musheev M., Schock F., Fornalczyk K., Ma D., Birk U. (2015). A transient ischemic environment induces reversible compaction of chromatin. Genome Biol..

[41] Cremer T., Cremer M., Cremer C. (2018). The 4D Nucleome: Genome Compartmentalization in an Evolutionary Context. Biochemistry.

[42] Roberts T.C. (2014). The MicroRNA Biology of the Mammalian Nucleus. Mol. Ther. Nucleic Acids.

[43] Fabian M.R., Sonenberg N., Filipowicz W. (2010). Regulation of mRNA Translation and Stability by microRNAs. Annu. Rev. Biochem..

[44] Leung A.K.L., Sharp P.A. (2012). Quantifying Argonaute Proteins In and Out of GW/P-Bodies: Implications in microRNA Activities. Adv. Exp. Med. Biol..

[45] Singh I., Contreras A., Cordero J., Rubio K., Dobersch S., Günther S., Jeratsch S., Mehta A., Krüger M., Graumann J. (2018). MiCEE is a ncRNA-protein complex that mediates epigenetic silencing and nucleolar organization. Nat. Genet..

[46] Leung A.K.L. (2015). The Whereabouts of microRNA Actions: Cytoplasm and Beyond. Trends Cell Biol..

[47] Sleeman J.P., Christofori G., Fodde R., Collard J.G., Berx G., Decraene C., Rüegg C. (2012). Concepts of metastasis in flux: The stromal progression model. Semin. Cancer Biol..

[48] Oskarsson T., Batlle E., Massagué J. (2014). Metastatic Stem Cells: Sources, Niches, and Vital Pathways. Cell Stem Cell.

[49] Allgayer H., Leupold J.H., Patil N. (2020). Defining the “Metastasome”: Perspectives from the genome and molecular landscape in colorectal cancer for metastasis evolution and clinical consequences. Semin. Cancer Biol..

[50] Hübner B., Lomiento M., Mammoli F., Illner D., Markaki Y., Ferrari S., Cremer M., Cremer T. (2015). Remodeling of nuclear landscapes during human myelopoietic cell differentiation maintains co-aligned active and inactive nuclear compartments. Epigenet. Chromatin.

[51] Lieberman-Aiden E., Van Berkum N.L., Williams L., Imakaev M., Ragoczy T., Telling A., Amit I., Lajoie B.R., Sabo P.J., Dorschner M.O. (2009). Comprehensive Mapping of Long-Range Interactions Reveals Folding Principles of the Human Genome. Science.

[52] Dekker J., Mirny L. (2016). The 3D Genome as Moderator of Chromosomal Communication. Cell.

[53] Bintu B., Mateo L.J., Su J.-H., Sinnott-Armstrong N.A., Parker M., Kinrot S., Yamaya K., Boettiger A.N., Zhuang X. (2018). Super-resolution chromatin tracing reveals domains and cooperative interactions in single cells. Science.

[54] Szabo Q., Jost D., Chang J.-M., Cattoni D.I., Papadopoulos G.L., Bonev B., Sexton T., Gurgo J., Jacquier C., Nollmann M. (2018). TADs are 3D structural units of higher-order chromosome organization inDrosophila. Sci. Adv..

[55] Otterstrom J., Castells-Garcia A., Vicario C., García P.A.G., Cosma M.P., Lakadamyali M. (2019). Super-resolution microscopy reveals how histone tail acetylation affects DNA compaction within nucleosomes in vivo. Nucleic Acids Res..

[56] Beliveau B.J., Boettiger A.N., Avendaño M.S., Jungmann R., McCole R.B., Joyce E.F., Kim-Kiselak C., Bantignies F., Fonseka C.Y., Erceg J. (2015). Single-molecule super-resolution imaging of chromosomes and in situ haplotype visualization using Oligopaint FISH probes. Nat. Commun..

[57] Mateo L., Murphy S.E., Hafner A., Cinquini I.S., Walker C.A., Boettiger A.N. (2019). Visualizing DNA folding and RNA in embryos at single-cell resolution. Nat. Cell Biol..

[58] Esa A., Edelmann P., Kreth G., Trakhtenbrot L., Amariglio N., Rechavi G., Hausmann M., Cremer C. (2000). Three-dimensional spectral precision distance microscopy of chromatin nanostructures after triple-colour DNA labelling: A study of the BCR region on chromosome 22 and the Philadelphia chromosome. J. Microsc..

[59] Shivashankar G. (2019). Mechanical regulation of genome architecture and cell-fate decisions. Curr. Opin. Cell Biol..

[60] Dreger M., Madrazo E., Hurlstone A., Redondo-Muñoz J. (2019). Novel contribution of epigenetic changes to nuclear dynamics. Nucleus.

[61] Nelson L.D., Bender C., Mannsperger H., Buergy D., Kambakamba P., Mudduluru G., Korf U., Hughes D., Van Dyke M.W., Allgayer H. (2012). Triplex DNA-binding proteins are associated with clinical outcomes revealed by proteomic measurements in patients with colorectal cancer. Mol. Cancer.

[62] Diederich B., Helle Ø., Then P., Carravilla P., Schink K.O., Hornung F., Deinhardt-Emmer S., Eggeling C., Ahluwalia B.S., Heintzmann R. (2020). Nanoscopy on the Chea(i)p. bioRxiv.

[63] Alix-Panabières C., Pantel K. (2021). Liquid Biopsy: From Discovery to Clinical Application. Cancer Discov..

[64] Keller L., Pantel K. (2019). Unravelling tumour heterogeneity by single-cell profiling of circulating tumour cells. Nat. Rev. Cancer.

[65] Anfossi S., Babayan A., Pantel K., Calin G.A. (2018). Clinical utility of circulating non-coding RNAs—An update. Nat. Rev. Clin. Oncol..

