# Supplementary material for: Tackling Tumour Cell Heterogeneity at the Super-Resolution Level in Human Colorectal Cancer Tissue
Source: Cancers (Basel). 2021 Jul 22;13(15):3692. doi: 10.3390/cancers13153692 (PMC8345115; doi:10.3390/cancers13153692)
Supplement: Supplementary file 1 [file cancers-13-03692-s001.zip › cancers-1310095-supplementary.pdf]

cancers

MDPI

Supplementary Materials: Tackling Tumour Cell Heterogeneity at the Super-Resolution Level in Human Colorectal Cancer Tissue

Fabian Lang, María F. Contreras-Gerenas, Márton Gelléri, Jan Neumann, Ole Kröger, Filip Sadlo, Krzysztof Berniak, Alexander Marx, Christoph Cremer, Hans-Achim Wagenknecht, and Heike Allgayer

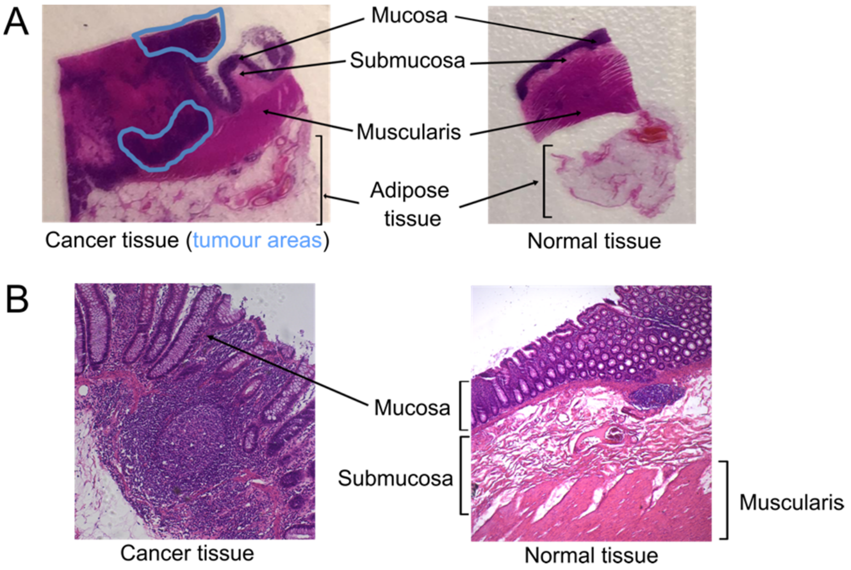

Figure S1. (A) Images of human colorectal tissue sections: Section of carcinoma tissue **(left)** and of the corresponding normal tissue **(right)**. Typical histological layers are depicted side by side for comparison. Blue areas correspond to the tumour areas (drawn by a pathologist) used for further imaging analysis. **(B)** Enlarged H&E image of a section of carcinoma **(left)** and normal **(right)** colorectal tissue. Magnification: 10x carcinoma tissue, 4x normal tissue. A magnification of 4x was used for the latter image with the aim to provide a clear visualisation of the different colorectal layers.

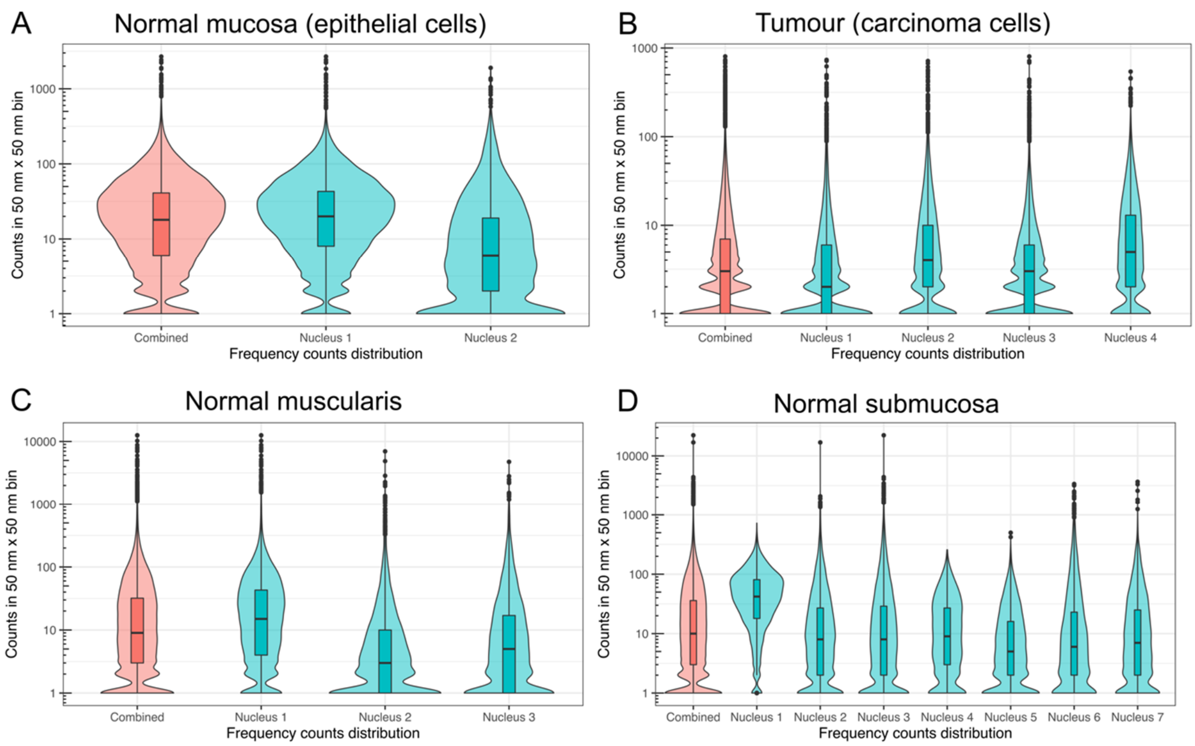

Figure S2. Violin + box plot representations of the localization (SMLM) signal histograms of normal and carcinoma human colorectal tissue nuclei. The first distribution in all graphs is the combination of the distributions of all of the replicates. Horizontal black lines in each of the box plots represent the median values. (N) denotes normal colorectal tissue. **(A)** Normal stromal cell nuclei within the submucosa-connective tissue region. **(B)** Normal epithelial cell nuclei located in the normal mucosa. **(C)** Normal muscle cell nuclei, muscularis layer. **(D)** Carcinoma cell nuclei, tumour tissue region.

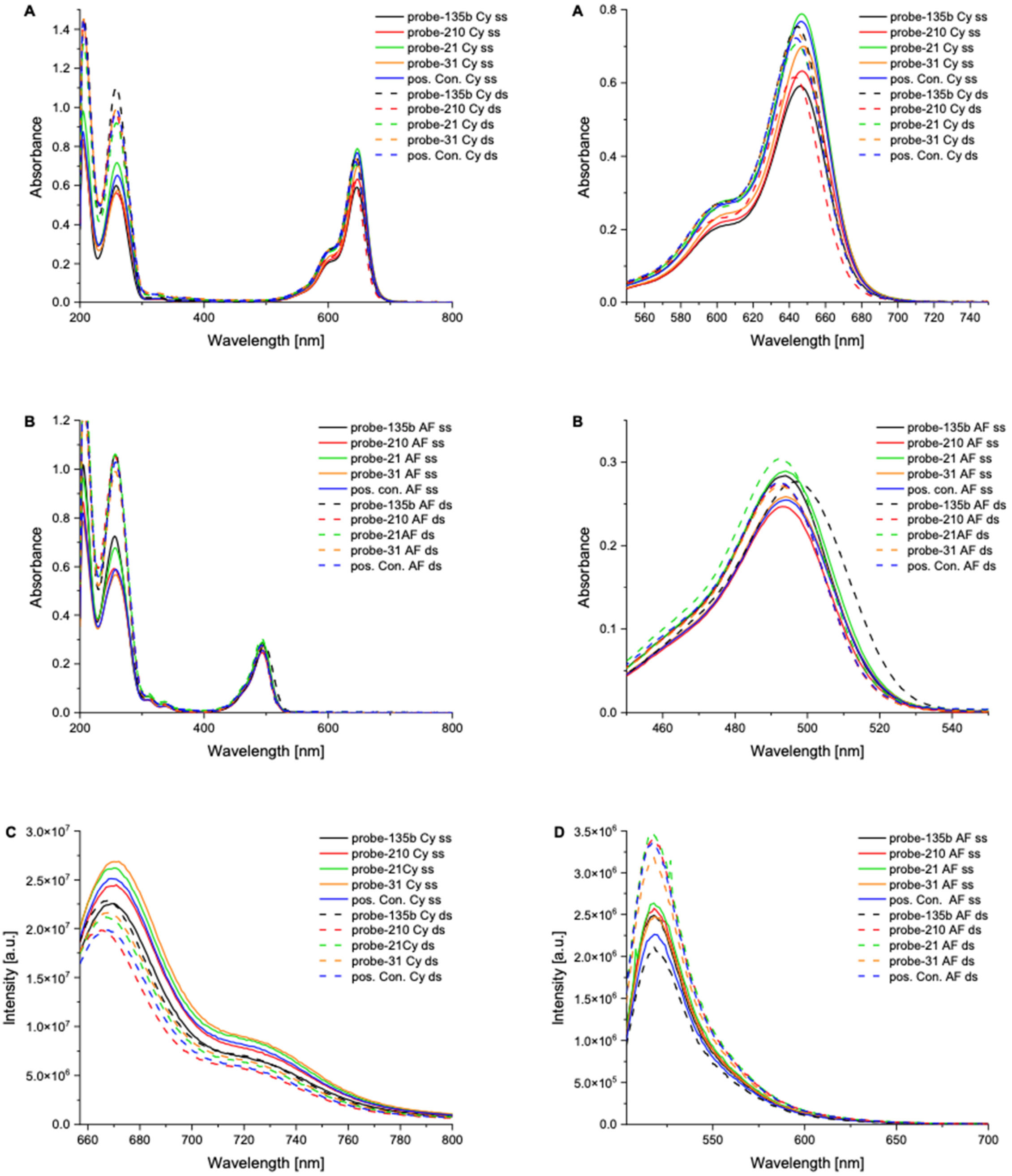

Figure S3. UV/Vis absorption (A and B) and fluorescence (C and D) of the synthetic DNA probes as single strands, and annealed in hybrids, with their target miRNA sequences; A and B on the right column correspond to enlargements of the peaks shown in spectra on the left (A and B). 2.5 μM DNA/RNA, 50 mM Na-P_i buffer, 250 mM NaCl, pH 7, λ_{exc}=647 nm for Cy5 probes, λ_{exc}=488 nm for AF488 probes.

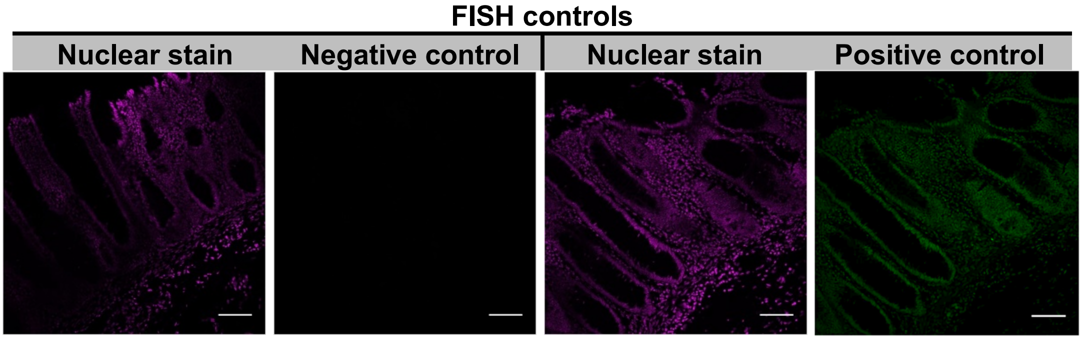

Figure S4. Positive and negative controls for the FISH staining. As negative control, a scrambled Scheme 6. snRNA probe. Nuclei were stained with Sytox Orange (purple), and the probe signal came from Cy5 (green), Scale bars: 100 µm.

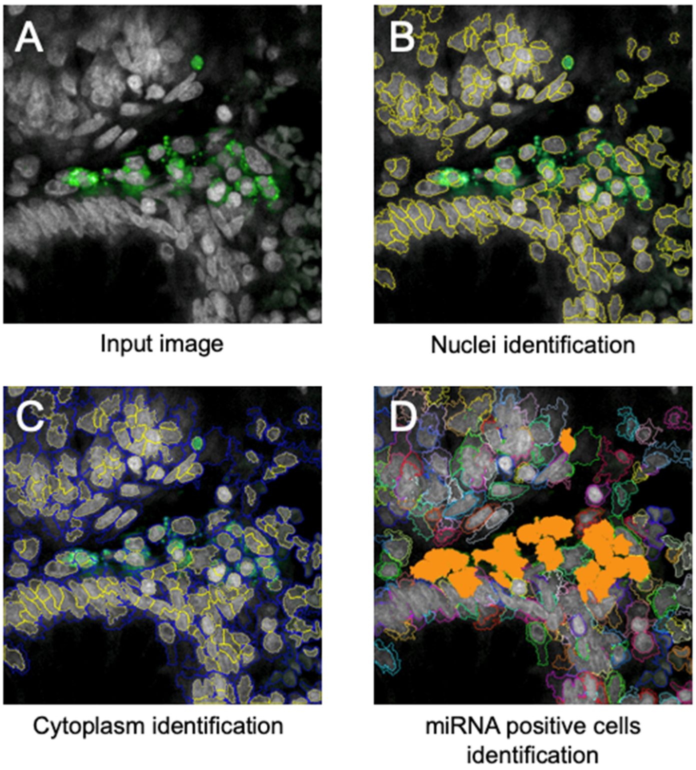

Figure S5. Identification of miRNA positive (miR+) cells using Columbus Software (PerkinElmer). **(A)** Representative image of a colorectal tissue sample after performing FISH with one of our DNA probes targeting a miRNA. **(B)** Nuclei were identified (yellow contours) based on the fluorescence signal from the DNA dye Sytox Orange. **(C)** An area surrounding each nucleus was drawn (blue contours), estimating the cytoplasm of each cell. **(D)** miR+ cells were identified as cells that presented an intensity above 5 on the miRNA channel (green) in their cytoplasmic region. In this figure, these cells are highlighted in orange areas.
